# *Plasmodium falciparum* erythrocyte membrane protein 1 variants induce cell swelling and disrupt the blood–brain barrier in cerebral malaria

**DOI:** 10.1084/jem.20201266

**Published:** 2021-01-25

**Authors:** Yvonne Adams, Rebecca W. Olsen, Anja Bengtsson, Nanna Dalgaard, Mykola Zdioruk, Sanghamitra Satpathi, Prativa K. Behera, Praveen K. Sahu, Sean E. Lawler, Klaus Qvortrup, Samuel C. Wassmer, Anja T.R. Jensen

**Affiliations:** 1 Centre for Medical Parasitology at Department of Immunology and Microbiology, Faculty of Health and Medical Sciences, University of Copenhagen, Copenhagen, Denmark; 2 Brigham and Women’s Hospital, Boston, MA; 3 Harvard Medical School, Boston, MA; 4 Department of Pathology, Ispat General Hospital, Rourkela, India; 5 Center for the Study of Complex Malaria in India, Ispat General Hospital, Rourkela, India; 6 Core Facility for Integrated Microscopy, Faculty of Health and Medical Sciences, University of Copenhagen, Copenhagen, Denmark; 7 Department of Infection Biology, London School of Hygiene and Tropical Medicine, London, UK

## Abstract

Cerebral malaria (CM) is caused by the binding of *Plasmodium falciparum*–infected erythrocytes (IEs) to the brain microvasculature, leading to inflammation, vessel occlusion, and cerebral swelling. We have previously linked dual intercellular adhesion molecule-1 (ICAM-1)– and endothelial protein C receptor (EPCR)–binding *P. falciparum* parasites to these symptoms, but the mechanism driving the pathogenesis has not been identified. Here, we used a 3D spheroid model of the blood–brain barrier (BBB) to determine unexpected new features of IEs expressing the dual-receptor binding PfEMP1 parasite proteins. Analysis of multiple parasite lines shows that IEs are taken up by brain endothelial cells in an ICAM-1–dependent manner, resulting in breakdown of the BBB and swelling of the endothelial cells. Via ex vivo analysis of postmortem tissue samples from CM patients, we confirmed the presence of parasites within brain endothelial cells. Importantly, this discovery points to parasite ingress into the brain endothelium as a contributing factor to the pathology of human CM.

## Teaser

*Plasmodium falciparum* parasites causing cerebral malaria express specific PfEMP1 proteins (group A^+CM^) on infected erythrocytes. Group A^+CM^ PfEMP1s allow entry into brain microvascular endothelial cells; this causes cell swelling and drives cerebral malaria pathology by disrupting the blood–brain barrier.

## Graphical Abstract

**Figure:**
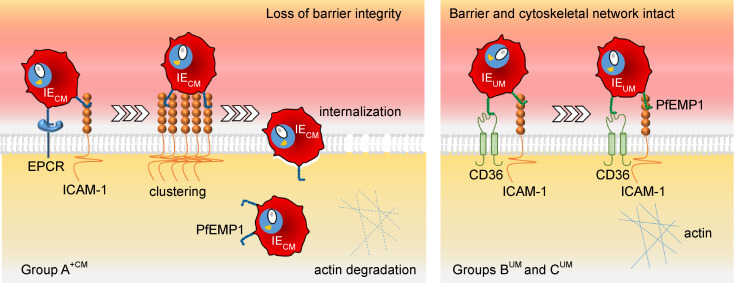

## Introduction

**Figure S1.**
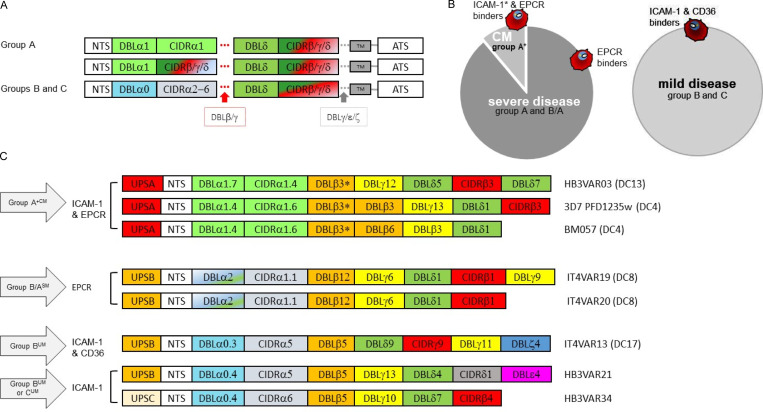
**PfEMP1 proteins are composed of different subtypes of DBL (α, β, γ, δ, ε, ξ, x) and CIDR (α, β, γ, δ) domains with different receptor specificity. (A)** Group B and C PfEMP1 predominantly have a four-domain structure, while the larger group A PfEMP1 proteins have additional DBL domains following the first or second DBL-CIDR domains. CIDR, cysteine-rich inter-domain region; ATS, acidic intracellular terminal segment. **(B)** CM constitutes a subset of severe disease cases and associates with group A^+CM^ IEs expressing dual receptor–binding PfEMP1s. ICAM-1–binding motif of such PfEMP1s here indicated by an asterisk (*) in the left circle. IEs associated with severe but noncerebral *P. falciparum* infection express ECPR binding group A^SM^ or B/A^SM^ PfEMP1s that do not bind ICAM-1 (left circle). IEs associated with mild *P. falciparum* infections express PfEMP1 that binds both ICAM-1 and CD36 or ICAM-1 only. Such group B^UM^ and group C^UM^ PfEMP1s do not carry a distinct ICAM-1–binding motif (right circle). **(C)** Domain structure of PfEMP1s expressed by *P. falciparum* parasite lines used in this study. CM associates with IEs expressing group A^+CM^ dual ICAM-1 and EPCR binding PfEMP1s; parasite lines expressing such PfEMP1s are HB3VAR03, 3D7 PFD1235w, and BM057. The ICAM-1–binding motif is indicated by an asterisk in their DBLβ (Lennartz et al., 2017). Non-cerebral SM associates with IEs expressing group B/A^SM^ (and A^SM^ shown in B) EPCR-binding PfEMP1s that do not bind ICAM-1. Domain cassette (DC) 8 is a chimeric gene between a group A and group B *var* gene. Parasite lines expressing group B/A^SM^ PfEMP1s are IT4VAR19 and IT4VAR20. UM associates with IEs that express group B^UM^ or C^UM^ PfEMP1 that binds both ICAM-1 and CD36 or ICAM-1. Parasite lines expressing group B^UM^ PfEMP1s are IT4VAR13 and HB3VAR21. The HB3VAR34 parasite line expresses a group C^UM^ PfEMP1. UPS, upstream promotor sequence; TM, transmembrane; NTS, N-terminal segment. Redrawn and modified from Jensen et al. (2020).

Approximately 60 different *Plasmodium falciparum* erythrocyte membrane protein 1 (PfEMP1) variants are encoded by each haploid genome of *P. falciparum,* allowing this human malaria parasite to undergo antigenic variation and evade the immune system. The *var* genes encoding PfEMP1 can be classed into groups A–E, depending on their chromosomal location and upstream promoter regions (Fig. S1 A; Gardner et al., 2002). The surface-expressed PfEMP1s and their Duffy-binding–like (DBL) and cysteine-rich inter-domain regions enable the parasite to attach infected erythrocytes (IEs) to different receptors on the endothelial lining, preventing passage of late-stage IEs through the spleen, thereby avoiding destruction (Weiss, 1990; Bachmann et al., 2009; reviewed in Hviid and Jensen, 2015). In severe disease, IE receptor adhesion leads to occlusion of the vessels. When this occurs in the brain, it can lead to sudden, rapid swelling of the brain, and death can result from respiratory failure due to brain stem herniation (Seydel et al., 2015; Newton et al., 1991; Crawley et al., 1998; Potchen et al., 2018). In the murine *Plasmodium chabaudi* model of malaria, IEs are observed perivascular to multiple organs, including the brain (Mota et al., 2000). These perivascular parasites were accompanied by gross morphological changes to the endothelial surface with microvilli-like projections and swelling of the endothelial cells (Mota et al., 2000). To date, there is limited information on the presence of *P. falciparum*–IEs beyond the blood–brain barrier (BBB), with only one postmortem study reporting the presence of IEs within the brain parenchyma (Pongponratn et al., 2003).

The endothelium can act as a nonprofessional phagocytic cell, actively taking up erythrocytes damaged by oxidative stress (Fens et al., 2010; Chang et al., 2018). An active role of the endothelium in clearing blood clots has been proposed; this process, also known as angiophagy, has been shown to govern the extravasation of clots beyond the vessels boundary (Grutzendler, 2013; Grutzendler et al., 2014). We hypothesized that *P. falciparum*–IEs associated with human cerebral malaria (CM) may trigger a response similar to cerebral microbleeds, resulting in the uptake of the parasites by brain endothelial cells. Since the identification of endothelial protein C receptor (EPCR)–binding parasites expressing group A and group B/A DC8 PfEMP1s and their association with severe malaria (SM; Moxon et al., 2013; Turner et al., 2013), parasites associated with severe disease can be further characterized to a subset causing CM (Fig. S1 B). Such *P. falciparum* CM parasites express a subgroup of group A PfEMP1s (group A^+CM^) that facilitates dual binding to host intercellular adhesion molecule-1 (ICAM-1) and EPCR (Lennartz et al., 2017). These group A^+CM^ PfEMP1s are characterized by the presence of a particular motif in their ICAM-1–binding sub-domain (DBLβ), which is absent in non–ICAM-1–binding EPCR–adherent IEs, and in DBLβ of ICAM-1–binding group B and C PfEMP1s (group B^UM^ and C^UM^; Fig. S1, B and C) associated with uncomplicated malaria (UM; Lennartz et al., 2017; Olsen et al., 2019; Turner et al., 2013).

## Results

### Group A^+CM^ IEs induce ICAM-1 clustering and are engulfed by endothelial cells

**Figure 1.**
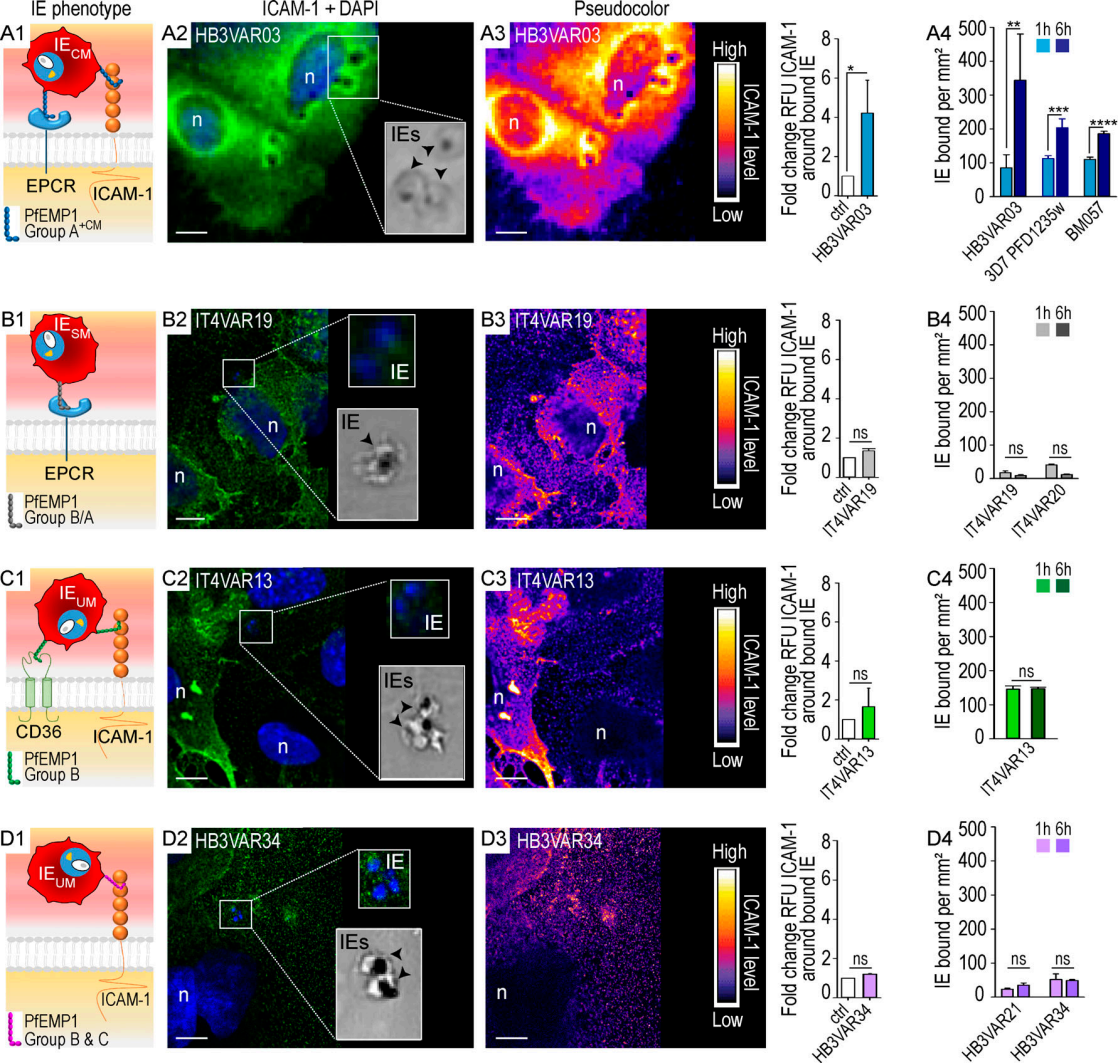
**Group A**^**+CM**^** IEs induce clustering of ICAM-1 on brain endothelial cells. (A1–A4) **IEs expressing group A^+CM^ ICAM-1 and EPCR dual-binding PfEMP1s with a specific ICAM-1–binding motif associated with CM. IE lines in this category include HB3VAR03-IEs, 3D7 PFD1235w-IEs, and BM057-IEs. **(B1–B4) **IEs expressing EPCR-binding group B/A^SM^ PfEMPs associated with SM that do not bind ICAM-1. IE lines in this category include IT4VAR19-IEs and IT4VAR20-IEs. **(C1–C4)** IEs expressing dual group B^UM^ ICAM-1– and CD36-binding PfEMP1s associated with UM lacking the motif found in group A^+CM^ PfEMP1s. IE line in this category is IT4VAR13-IEs. **(D1–D4)** IEs expressing group B^UM^ or C^UM^ PfEMP1s associated with UM that do bind ICAM-1, but lack the motif found in group A^+CM^ PfEMP1s. IE lines in this category include HB3VAR21-IEs and HB3VAR34-IEs. See also Fig. S1 and Table 1. **(A1–D1)** Schematic of the four different receptor binding IE phenotypes (group A^+CM^, group B/A^SM^, group B^UM^, and group C^UM^) used in this study. **(A2 and B2–D2)** Wide-field (A2) and confocal images (B2–D2) of hCMEC/D3 brain microvascular cells incubated with IEs. ICAM-1 is green (FITC), and DAPI nuclei stain is blue. The bottom insets of each panel show brightfield images of IEs (black arrows) present in the framed box to the left. Top insets are an enlargement of the framed box. Blue staining in the top insets shows the presence of parasite nuclei. The images are representative of at least three independent experiments. Scale bars, 10 µm. **(A2)** hCMEC/D3 cells incubated with dual ICAM-1– and EPCR-binding group A^+CM^ (HB3VAR03) IEs show ICAM-1 (green) clustered around base of the IEs. hCMEC/D3 cells incubated with (B2) EPCR-binding group B/A^SM^ (IT4VAR19), (C2) dual ICAM-1– and CD36-binding group B^UM^ (IT4VAR13), and (D2) ICAM-1–binding group C^UM^ (HB3VAR34) IEs do not induce clustering of ICAM-1. **(A3–D3)** Pseudo-coloring of the images in A2–D2. As indicated by the color bar, areas of high ICAM-1 levels are white and yellow, while areas with low ICAM-1 levels are dark blue. Scale bars, 10 µm. The graph to the right of each pseudo-colored image shows quantification of the ICAM-1 (RFU) clustering around bound IEs as compared with control (i.e., hCMEC/D3 cells incubated with noninfected erythrocytes). Calculations were based on a minimum of three independent experiments with at least 50 IEs counted per experiment. Shown are mean values ± SD; statistical significance was calculated using an unpaired *t* test (*, P ≤ 0.001; ns, not significant). **(A3)** Group A^+CM^ IEs (HB3VAR03) recruit or induce ICAM-1 clustering (evidenced by white, yellow, and red colors) by contrast to (B3) group B/A^SM^ (IT4VAR19), (C3) group B^UM^ (IT4VAR13), and (D3) group C^UM^ (HB3VAR34) IEs. n, nuclei. Images were processed with Fiji. **(A4–D4)** Binding (adhesion) of IEs to hCMEC/D3 cells at 1 h (lighter color) and 6 h (darker color). Shown are mean values ± SD of a minimum of three independent experiments conducted in duplicate. Statistical analysis was performed using an unpaired *t* test (**, P = 0.02; ***, P = 0.009; ****, P < 0.001; and ns, not significant). ICAM-1– and ECPR-binding group A^+CM^ IEs are HB3VAR03, 3D7 PFD1235w, and BM057 (A4); EPCR-binding group B/A^SM^ IEs are IT4VAR19 and IT4VAR20 (B4); ICAM-1– and CD36-binding group B^UM^ IE is IT4VAR13 (C4); and ICAM-1–binding group B^UM^ and C^UM^ IEs are HB3VAR21 and HB3VAR34 (D4), respectively. ctrl, control.

**Figure S2.**
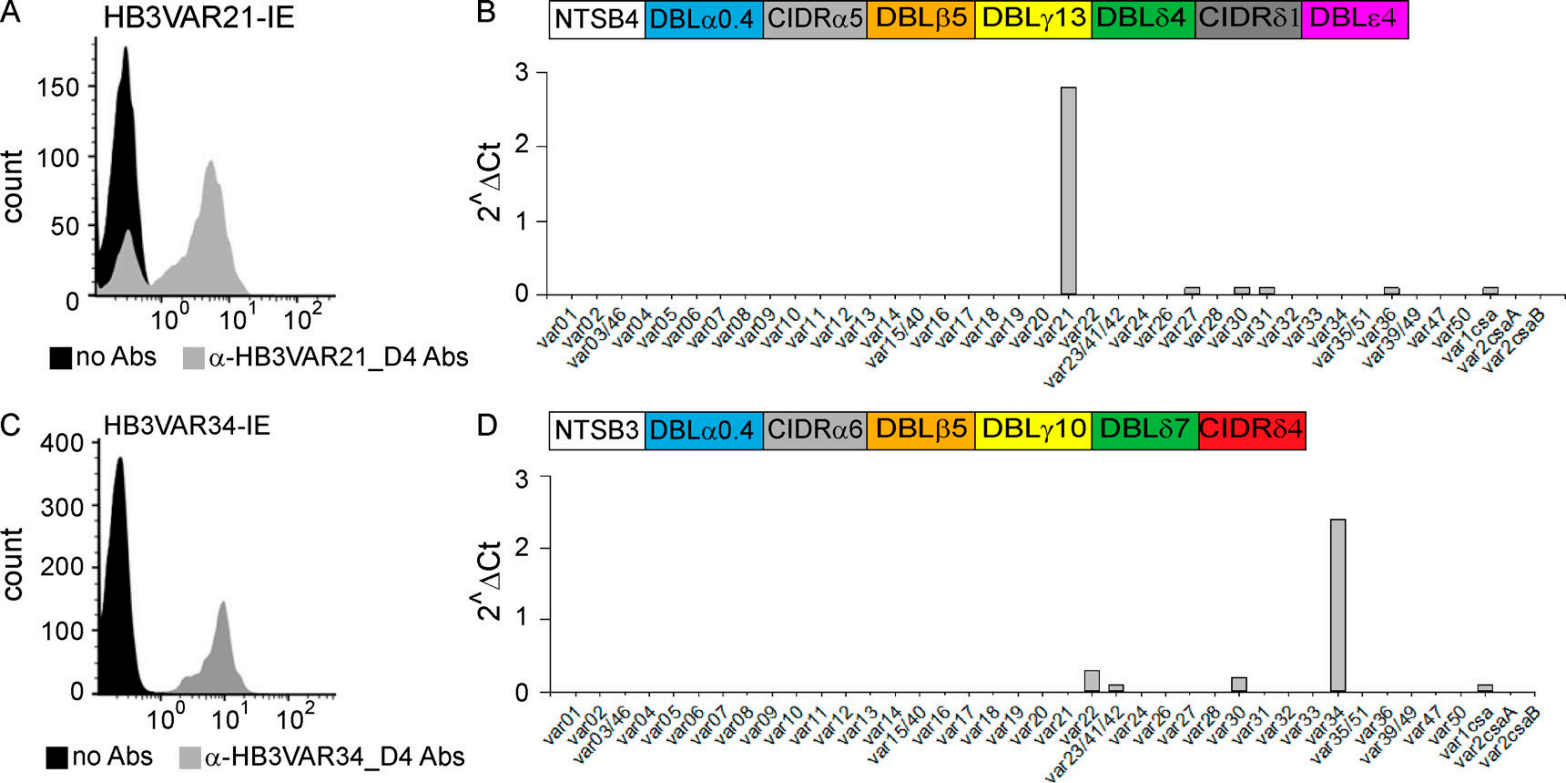
***var* gene and PfEMP1 expression profiles of HB3VAR21 and HB3VAR34 parasite lines. (A)** Ethidium bromide–stained group B^UM^ HB3VAR21-IE with (gray histogram) and without (black histogram) rat anti-HB3VAR21_DBLβ_D4 antiserum. **(B)** Transcript level of each *var* gene of HB3VAR21-IE relative to control gene (*seryl-tRNA synthetase*). The domain architecture of the dominant expressed PfEMP1 (HB3VAR21 or KOB63129.1) is shown. **(C)** Ethidium bromide–stained group C^UM^ HB3VAR34-IE with (gray histogram) and without (black histogram) rat HB3VAR34_DBLβ_D4 antiserum. **(D)** Transcript level of each *var* gene of HB3VAR34-IE relative to control gene (*seryl-tRNA synthetase*). The domain architecture of the dominant expressed PfEMP1 (HB3VAR34 or KOB58843) is shown. The data represent a minimum of three independent experiments conducted in duplicate. Abs, antibodies.

**Figure 2.**
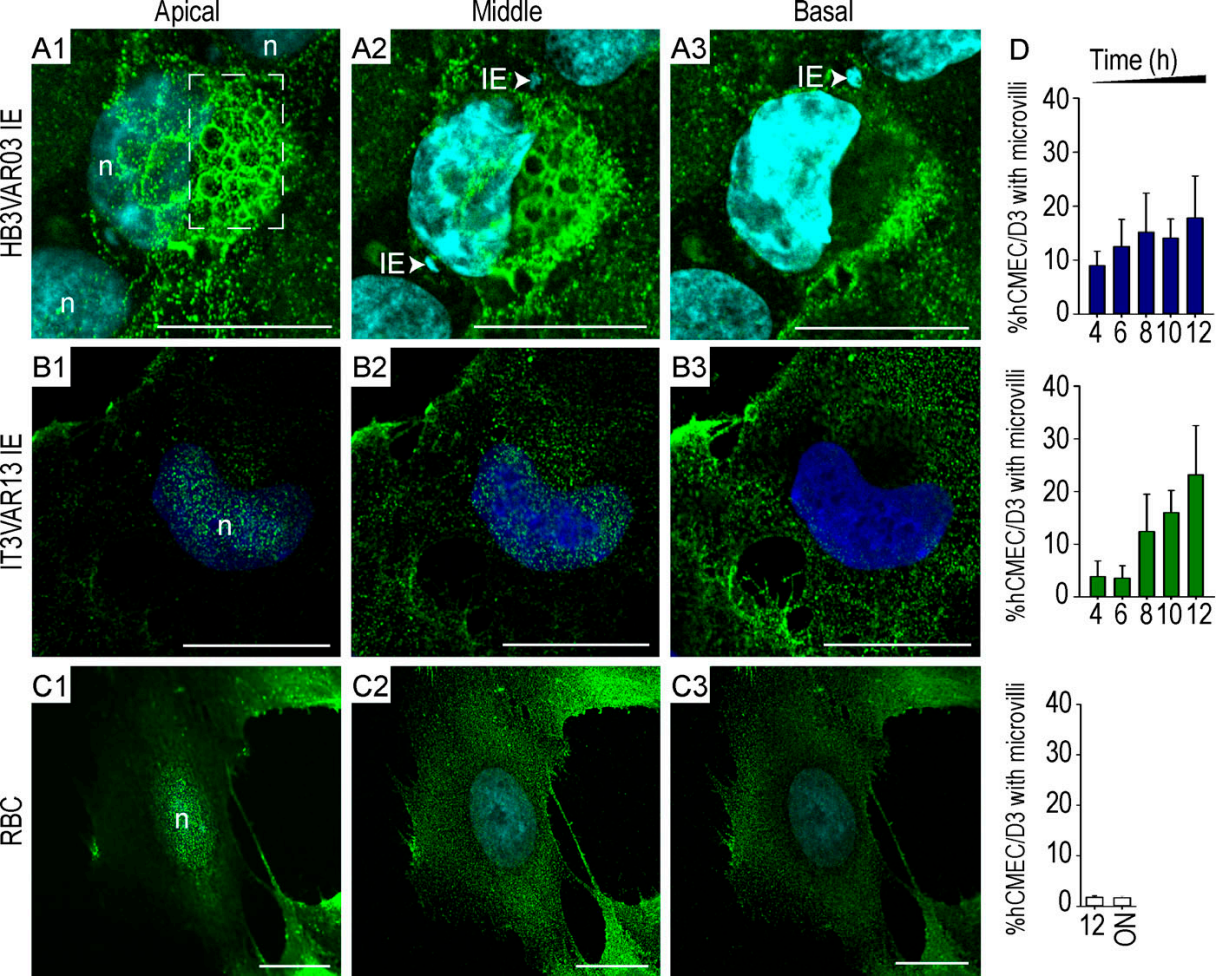
**ICAM-1–enriched microvilli and transmigratory ring/docking structures on brain hCMEC/D3 endothelial cells.** **(A–C)** hCMEC/D3 brain endothelial cells were incubated with parasites representative of (A) group A^+CM^ IEs (HB3VAR03), (B) group B^UM^ IEs (IT4VAR13), or (C) noninfected erythrocytes (RBC indicates RBC controls). ICAM-1 expression at the apical, middle, and basal surfaces of hCMEC/D3 cells were imaged using confocal microscopy. ICAM-1 is green (FITC), and DAPI nuceli stain is blue. n denotes the nuclei and is annotated in apical images. **(A)** The group A^+CM^ IEs (HB3VAR03) induce ICAM-1–enriched circular ring/docking structures of different sizes ranging from 1.6 to 3.7 µm. White arrows in A2 and A3 point to HB3VAR03-IEs. **(A–C)** The images are representative of a minimum of three independent experiments. Scale bars, 20 µm. **(D)** Percentage of hCMEC/D3 endothelial cells covered with ICAM-1–enriched microvilli following incubation with HBVAR03-IEs, IT4VAR13-IEs, or RBC controls. ON, overnight co-culture. All graphs show mean values ± SD for 200 cells per condition, for a minimum of three independent experiments conducted in duplicate. See also Fig. S3.

**Figure 3.**
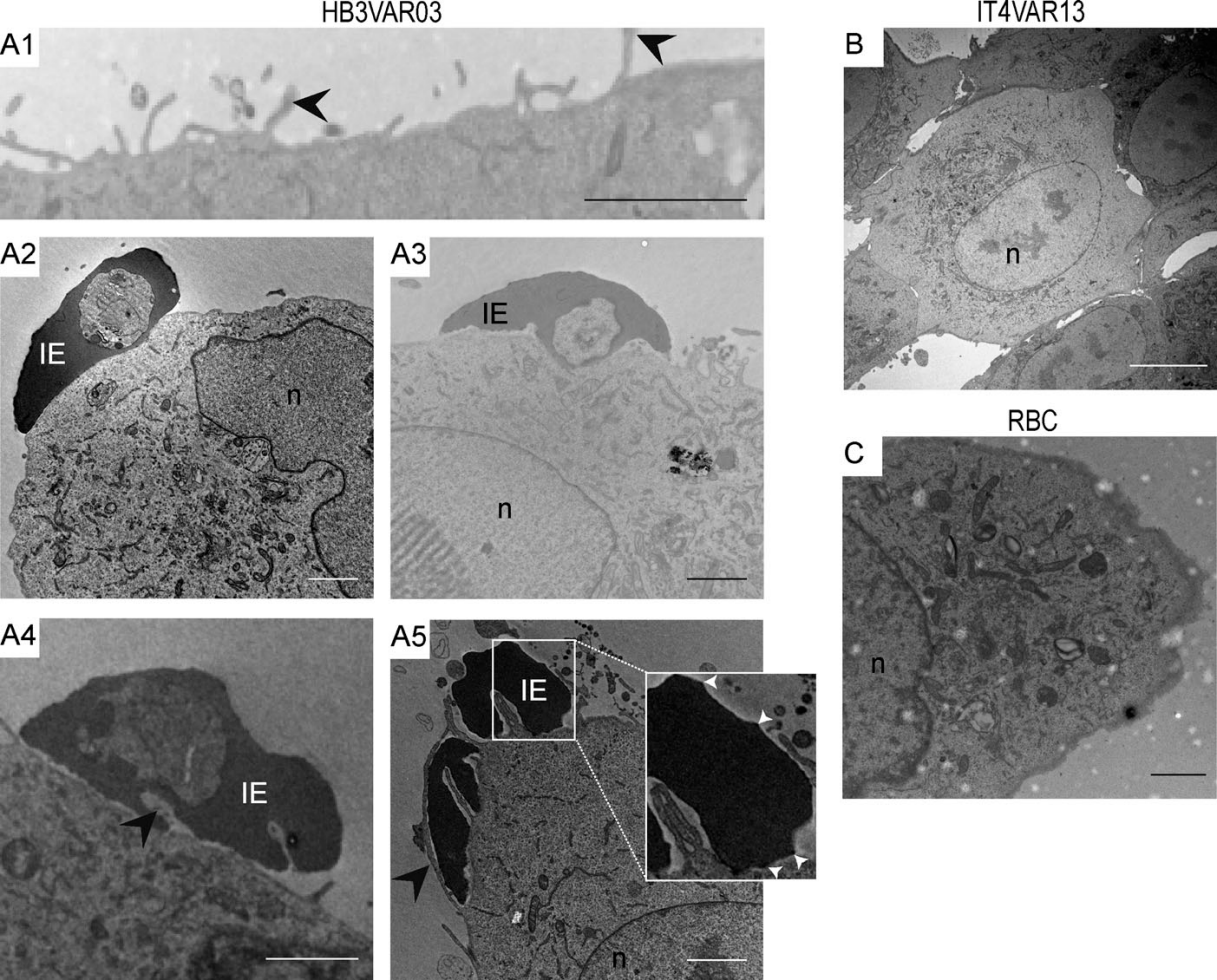
**Group A^+CM^ IEs induced altered morphology of endothelial cells.** **(A–C)** Electron microscopy images of brain microvascular cells (hCMEC/D3) co-cultured with (A) group A^+CM^ HB3VAR03-IEs, (B) group B^UM^ IT4VAR13-IEs, and (C) RBC controls (RBC). The images are representative of four independent experiments. Scale bars, A1–A5, 2 µm; B and C scale bars, 10 µm. **(A1)** Microvilli on hCMEC/D3 co-cultured with HB3VAR03-IE. **(A2 and A3)** Adherent HB3VAR03-IE on surface of hCMEC/D3. **(A3)** HB3VAR03-IE interacting with endothelial surface. **(A4)** Adherent HB3VAR03-IE showing invaginations of cell membrane, “pinching” microvilli (black arrowhead) protruding from endothelial membrane. **(A5)** Microvilli extending around an HB3VAR03-IE (black arrowhead) with electron-dense knobs seen around the IE membrane. Inset shows knobs (white arrowhead) at larger magnification. n, nuclei.

**Figure S3.**
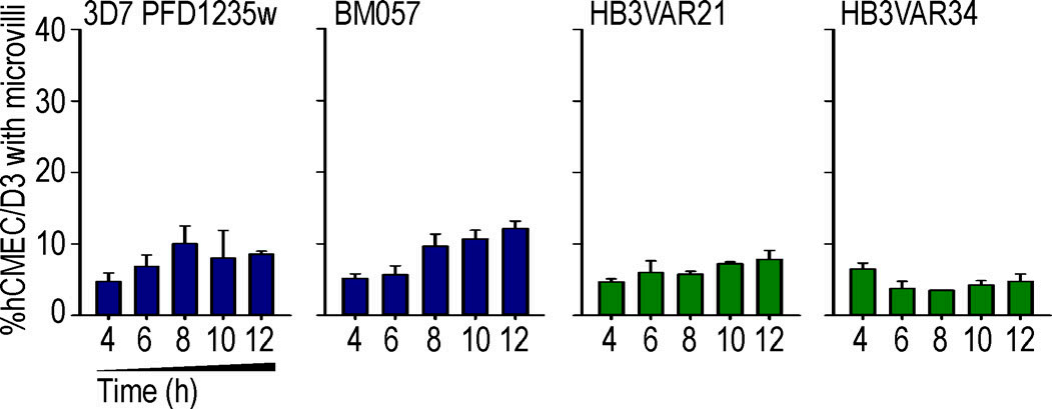
**ICAM-1–enriched microvilli on brain hCMEC/D3 endothelial cells.** Percentage of hCMEC/D3 endothelial cells covered with ICAM-1–enriched microvilli following incubation with group A^+CM^ (3D7 PFD1235w and BM057), group B^UM^ (HB3VAR21), or group C^UM^ (HBVAR34) IEs. Shown are mean values ± SD for a minimum of three independent experiments conducted in duplicate.

**Figure 4.**
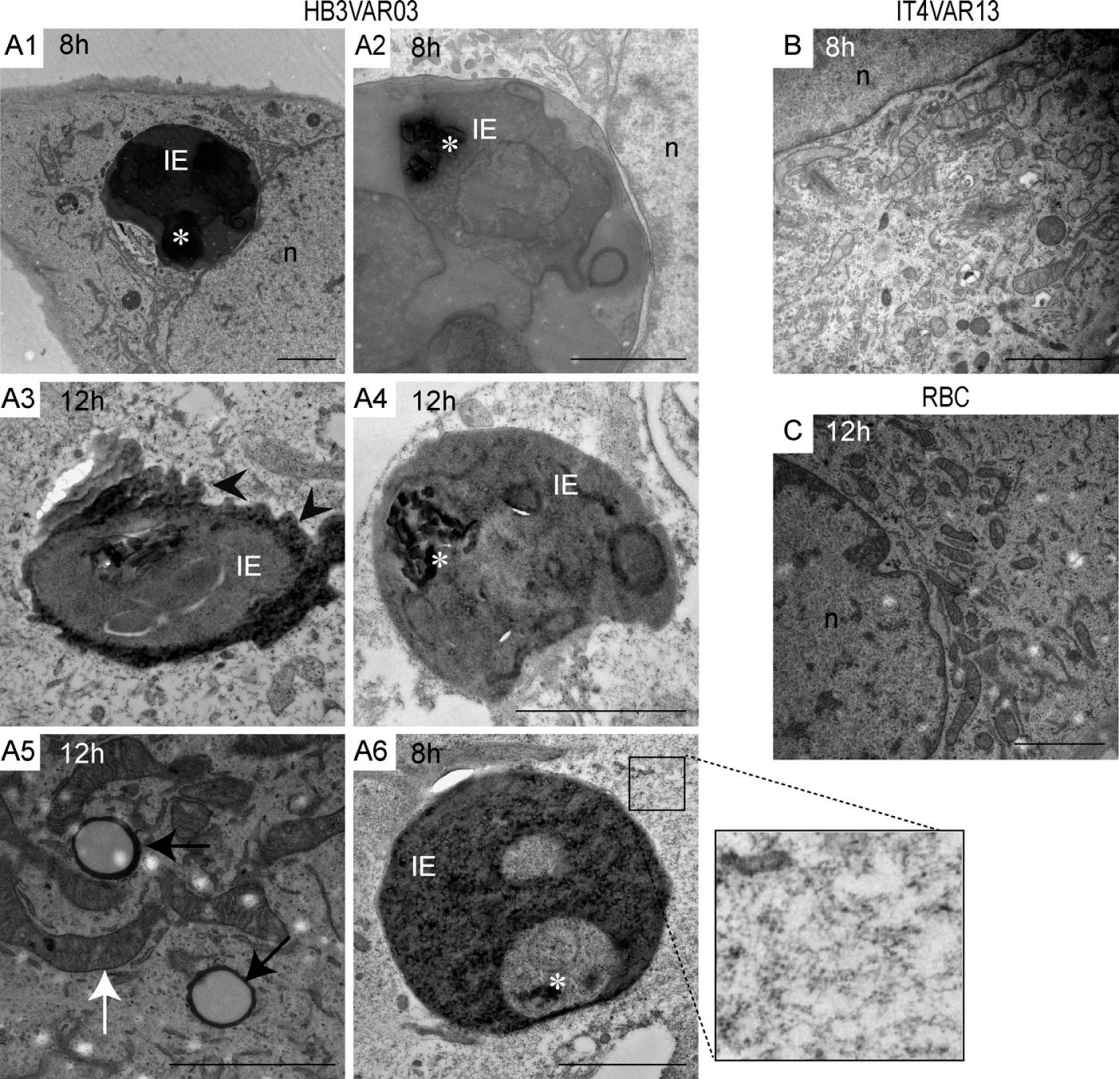
**Brain microvascular endothelial cells internalize *P. falciparum*–IEs. (A–C)** Electron microscopy images of brain microvascular cells (hCMEC/D3) co-cultured with (A) group A^+CM^ HB3VAR03-IEs, (B) group B^UM^ IT4VAR13-IEs, or (C) RBC controls (RBC). The images are representative of four independent experiments. Scale bars, all images, 2 µm. **(A1–A6)** hCMEC/D3 with internalized HB3VAR03 *P. falciparum*–IE at 8 and 12 h. n, nucleus of endothelial cell. Parasite hemozoin is indicated by a white asterisk (*) in A1–A4 and A6. **(A3)** Black arrowheads point to shrunken and ruffled membrane. **(A5)** Black arrows point to secretory pods associated with release of von Willebrand factor. White arrow points to one of several mitochondria. **(A6)** Inset shows enlargement of hCMEC/D3 showing aggregated cytoplasm indicative of actin stress fibers.

To investigate endothelial responses to *P. falciparum* parasites with defined adhesion phenotypes (Fig. 1, A1–D1; and Fig. S1), we coincubated human brain microvascular endothelial cells (hCMEC/D3) with dual ICAM-1– and EPCR-binding group A^+CM^ (HB3VAR03) IEs, dual ICAM-1– and CD36-binding group B^UM^ (IT4VAR13) IEs, single ICAM-1–binding group C^UM^ (HB3VAR34) IEs, or single EPCR-binding group B/A^SM^ IEs expressing the DC8 variant IT4VAR19 (Turner et al., 2013; Gillrie et al., 2015; Avril et al., 2013). IEs were incubated for 1–12 h and endothelial cells subsequently stained for ICAM-1 expression. A distinct pattern of ICAM-1 stain was observed surrounding the ICAM-1/EPCR–binding group A^+CM^ IEs (Fig. 1, A2 and A3), but not around the EPCR-binding group B/A^SM^ IEs (Fig. 1, B2 and B3), group B^UM^ ICAM-1/CD36–binding group IEs (Fig. 1, C2 and C3), or the ICAM-1–binding group C^UM^ IEs (Fig. 1, D2 and D3). The number of bound IEs was also observed to significantly increase over time (1 vs. 6 h) for erythrocytes infected by three different lines (HB3VAR03, 3D7 PFD1235w, and BM057) of group A^+CM^ parasites (Fig. 1 A4 and Table 1), while no significant change was observed for erythrocytes infected by group B^UM^ and C^UM^ (IT4VAR13, HB3VAR21, and HB3VAR34), nor group B/A^SM^ parasites (IT4VAR19 and IT4VAR20; Fig. 1, B4–D4; and Fig. S2). Remarkably, endothelial cells exposed to group A^+CM^ IEs (HB3VAR03) showed many circular gaps ranging from 1.6 to 3.7 µm in diameter (Fig. 2 A). These ICAM-1–enriched circular structures were observed on multiple cells, with some colocalizing with IEs (Fig. 1 A2; Fig. 2, A2 and A3; and Fig. 6 I), and the structures that we termed rings/docking structures extended from the apical surface, toward the basal layer (Fig. 2, A1–A3). The group B^UM^ (IT4VAR13) IEs and red blood cell controls did not induce ring/docking structures (Fig. 2, B1–C3). Multiple ICAM-1–enriched protrusions or microvilli were also observed on the endothelial cell surface (Fig. 2 and Fig. 3), independent of the PfEMP1 variant expressed by the IEs (Fig. 2 D and Fig. S3). Other endothelial cells possessed microvilli without adherent IEs (Fig. 2 B), suggesting that the microvilli production did not appear to depend on cell-to-cell interactions. Further analysis via electron microscopy revealed gross structural changes to the apical membrane of hCMEC/D3 cells after co-culture with group A^+CM^ HB3VAR03-IEs (Fig. 3 A). The endothelial cell membrane had extended into long, microvilli–finger-like projections observed interacting with surface-bound IEs (Fig. 3, A2–A4). In some instances, the IE membrane showed invaginations (“pinching”) around endothelial microvilli (Fig. 3, A3 and A4), while in others, microvilli were observed surrounding the IE (Fig. 3 A5 and inset) similar to dome formation observed in leukocyte transmigration (Phillipson et al., 2008). The most striking and surprising observation was the presence of intact IEs within the endothelial cells (Fig. 4, A1–A4 and A6). Multiple mitochondria were also observed as well as secretory pods defined by their size and shape, along with the granularity inside their membrane (Fig. 4 A5). Weibel–Palade bodies were not obvious, but the presence of secretory pods suggest they may have been exhausted. Extended co-culture (10 h to overnight) also showed readily identifiable group A^+CM^ late trophozoite IEs within endothelial cells. However, the surrounding red blood cell membrane was highly degraded, with a mottled pattern, and the endothelial cytoplasm showed evidence of actin fibrils, indicative of cellular stress (Fig. 4 A6 and inset). Control experiments with group B^UM^ (IT4VAR13) IEs and noninfected erythrocytes performed in parallel did not show erythrocyte internalization within hCMEC/D3 cells after 8 or 12 h, and the cytoplasm of the exposed hCMEC/D3 appeared normal (Fig. 3, B and C; and Fig. 4, B and C).

**Table 1.** *P. falciparum* isolates used to generate experimental data

Isolate	PfEMP1	Domain subtype	Motif	Adhesion phenotype	*var* group
HB3	HB3VAR03	DBLβ3_D4	Yes	ICAM-1, EPCR, PECAM-1^a^	A
3D7	PFD1235w^b^	DBLβ3_D4	Yes	ICAM-1, EPCR^c^	A
BM057	JN037695	DBLβ3_D4	Yes	ICAM-1, EPCR^d^	A
HB3	HB3VAR21^e^	DBLβ5_D4	No	ICAM-1^f^	B
HB3	HB3VAR34^g^	DBLβ5_D4	No	ICAM-1^f^	C
IT4	IT4VAR13^h^	DBLβ5_D4	No	ICAM-1, CD36^i^	B
IT4	IT4VAR19	DBLβ12_D4	No	EPCR^j^	B/A
IT4	IT4VAR20	DBLβ12_D4	No	EPCR^j^	B/A

a Data from Lennartz et al. (2017) and Joergensen et al. (2010).

b Also known as PF3D7_0425800.

c Data from Joergensen et al. (2010), Bengtsson et al. (2013), and Lennartz et al. (2017).

d Data from Bengtsson et al. (2013) and Lennartz et al. (2017).

e Also known as KOB63129.1.

f Data from Olsen et al. (2019).

g Also known as KOB58843.

h Also known as ABM88750.

i Data from Janes et al. (2011).

j Data from Turner et al. (2013).

### Internalization is mediated by PfEMP1::ICAM-1 interactions

**Figure 5.**
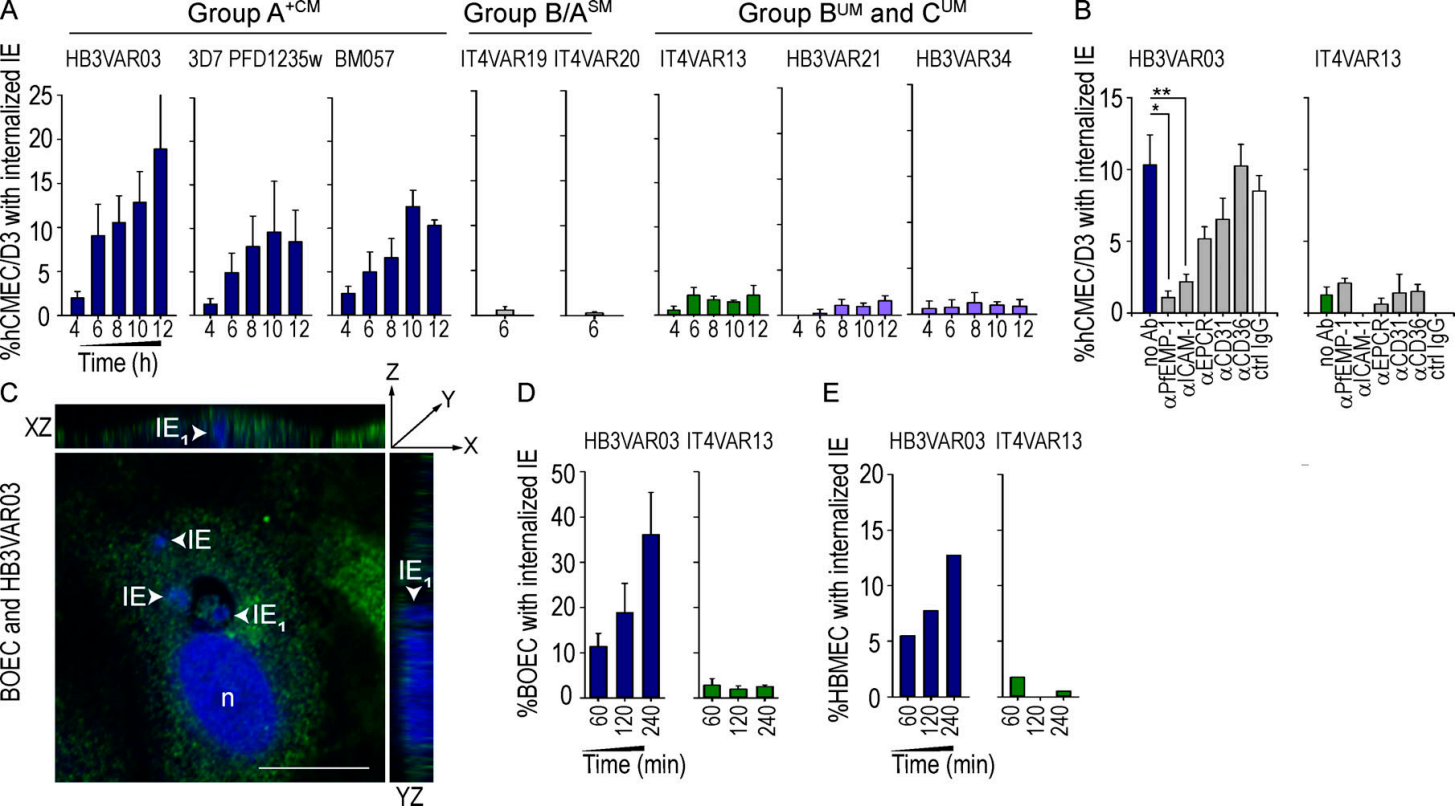
**PfEMP1– and ICAM-1–dependent internalization of *P. falciparum*–IE. (A)** Brain microvascular endothelial cells (hCMEC/D3) with internalized group A^+CM^ (HB3VAR03, 3D7 PFD12352, and BM057), group B/A^SM^ (IT4VAR19 and IT4VAR20), group B^UM^ (IT4VAR13 and HB3VAR34), and group C^UM^ (HB3VAR34) IEs (Table 1 and Fig. S1) after 4–12 h co-culture. All graphs show mean values ± SD for 200 cells per condition for a minimum of three independent experiments conducted in duplicate. **(B)** Internalization of IEs depends on the PfEMP1 sub-type expressed by the parasite isolate and on ICAM-1. Internalization of HB3VAR03-IEs and IT4VAR13-IEs was assessed with homologous αPfEMP1 (αHB3VAR03_D4, αIT4VAR13_D4 at 1:100), αICAM-1 (clone 15.2, 10 µg/ml), αEPCR (10 µg/ml), αCD31 (10 µg/ml), αCD36 (10 µg/ml), or control (ctrl) IgG (10 µg/ml) antibody. Graphs show mean values ± SD of a minimum of three independent experiments conducted in duplicate. Statistical analysis was done using one-way ANOVA with Tukey’s multiple comparison test (*, P < 0.001; **, P = 0.005). Only significant findings are shown. **(C)** Internalization of HB3VAR03-IEs into BOECs in monolayers was visualized using confocal microscopy. The image shows a representative orthogonal view of HB3VAR03-IEs (white arrowheads) surrounded by ICAM-1. ICAM-1 is green (FITC), and DAPI nuceli stain is blue. n denotes the nuclei of the BOEC. Scale bar, 20 µm. **(D)** Human BOECs and (E) human brain microvascular endothelial cells (HBMECs) with internalized group A^+CM^ (HB3VAR03) and group B^UM^ (IT4VAR13) IEs. The percentages of cells (hCMEC/D3, BOEC, and HBMEC) with internalized IEs (A, B, D, and E) were quantified by wide-field microscopy. Data in D show the mean of three independent experiments, while data in E show the mean values ± SD of two independent experiments conducted in duplicate.

Quantification of *P. falciparum* internalization by confocal microscopy showed that three different group A^+CM^ lines (HB3VAR03-IEs, 3D7 PFD1235w-IEs, and BM057-IEs; Table 1 and Fig. S1) were internalized by hCMEC/D3 cells in a time-dependent manner, while three group B^UM^ and C^UM^ lines (IT4VAR13-IEs, HB3VAR21-IEs, and HB3VAR34-IEs) and the two group B/A^SM^ lines (IT4VAR19-IEs and IT4VAR20-IEs) entered the hCMEC/D3 cells at a very low rate (Fig. 5 A). We next investigated the role of ICAM-1 in IE uptake by coincubating the hCMEC/D3 monolayers with anti–ICAM-1 antibody (clone 15.2) before the addition of IEs for 8 h. The anti–ICAM-1 antibody inhibited the internalization of group A^+CM^ HB3VAR03-IEs (P = 0.005), while internalization of group B^UM^ IT4VAR13-IEs remained unaltered and <2.5% (Fig. 5 B). Adding anti-EPCR and anti-CD31 antibodies showed a nonsignificant reduction in the internalization of the dual ICAM-1/EPCR–binding HB3VAR03-IEs (P = 0.36 and P = 1, respectively). *P. falciparum*–IEs were then coincubated with homologous antibody generated against the ICAM-1–binding DBLβ domain of both parasite isolates. The addition of the homologous anti-PfEMP1 DBLβ antibody significantly reduced the internalization of the group A^+CM^ HB3VAR03-IEs (P < 0.001), but not group B^UM^ IT4VAR13-IEs (Fig. 5 B). Collectively, these data show group A^+CM^ IEs enter endothelial cells in an ICAM-1– and PfEMP1-dependent manner.

### Internalization of group A^+CM^ IEs into blood outgrowth endothelial cells (BOECs) and human brain microvascular cells (HBMECs)

To assess whether other cell types engulf IEs, we used BOECs isolated from a European donor as described in Ecklu-Mensah et al. (2018). BOECs constitute a subpopulation of human endothelial cells, found in peripheral blood; they express multiple receptors including ICAM-1 and EPCR, and support adhesion of IEs (Ecklu-Mensah et al., 2018). We also used primary HBMECs to assess whether IEs can enter cells other than hCMEC/D3 (Lennartz et al., 2017). Similar to co-cultures of IEs and hCMEC/D3, group A^+CM^ (HB3VAR03) IEs, but not group B^UM^ (IT4VAR13) IEs, enter BOEC (Fig. 5, C and D) and HBMEC (Fig. 5 E). The more angiogenic BOEC (Fig. 5 D) displayed a higher level and faster uptake of group A^+CM^ IEs than hCMEC/D3 (Fig. 5 A) and HBMEC (Fig. 5 E). This may be due to their retention of a proangiogenic phenotype (Dauwe et al., 2016). These data support the findings that IEs can enter multiple cell types, and more importantly, it is not a general property of IEs, but restricted to a specific subset of group A PfEMP1s associated with CM.

### CM-associated IEs induce gross changes in an in vitro three-dimensional (3D) BBB model

**Figure S4.**
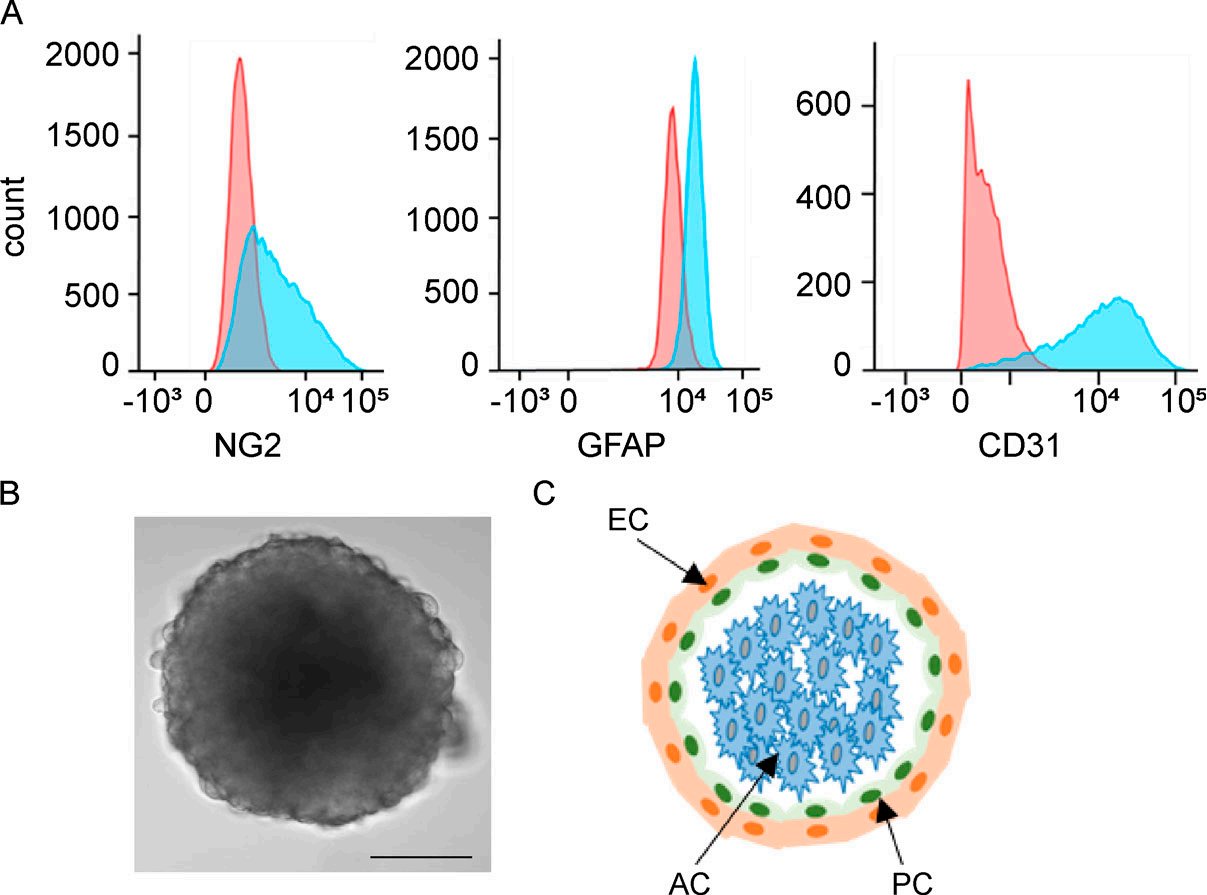
**3D BBB spheroids are composed of three different cell types. (A)** FACS histogram showing pericytes (NG2, neural/glial antigen 2), astrocytes (GFAP, glial fibrillary acidic protein), and human cerebral microvascular endothelial cells (hCMEC/D3 and CD31). The data are a representative FACS plot from two independent experiments conducted in duplicate. **(B)** Representative brightfield image of BBB spheroid. Scale bar, 100 µm. **(C)** Schematic of spheroid with a core comprised of astrocytes (AC), bound by pericytes (PC), and surrounded by endothelial cells (EC) at the outer surface.

**Figure 6.**
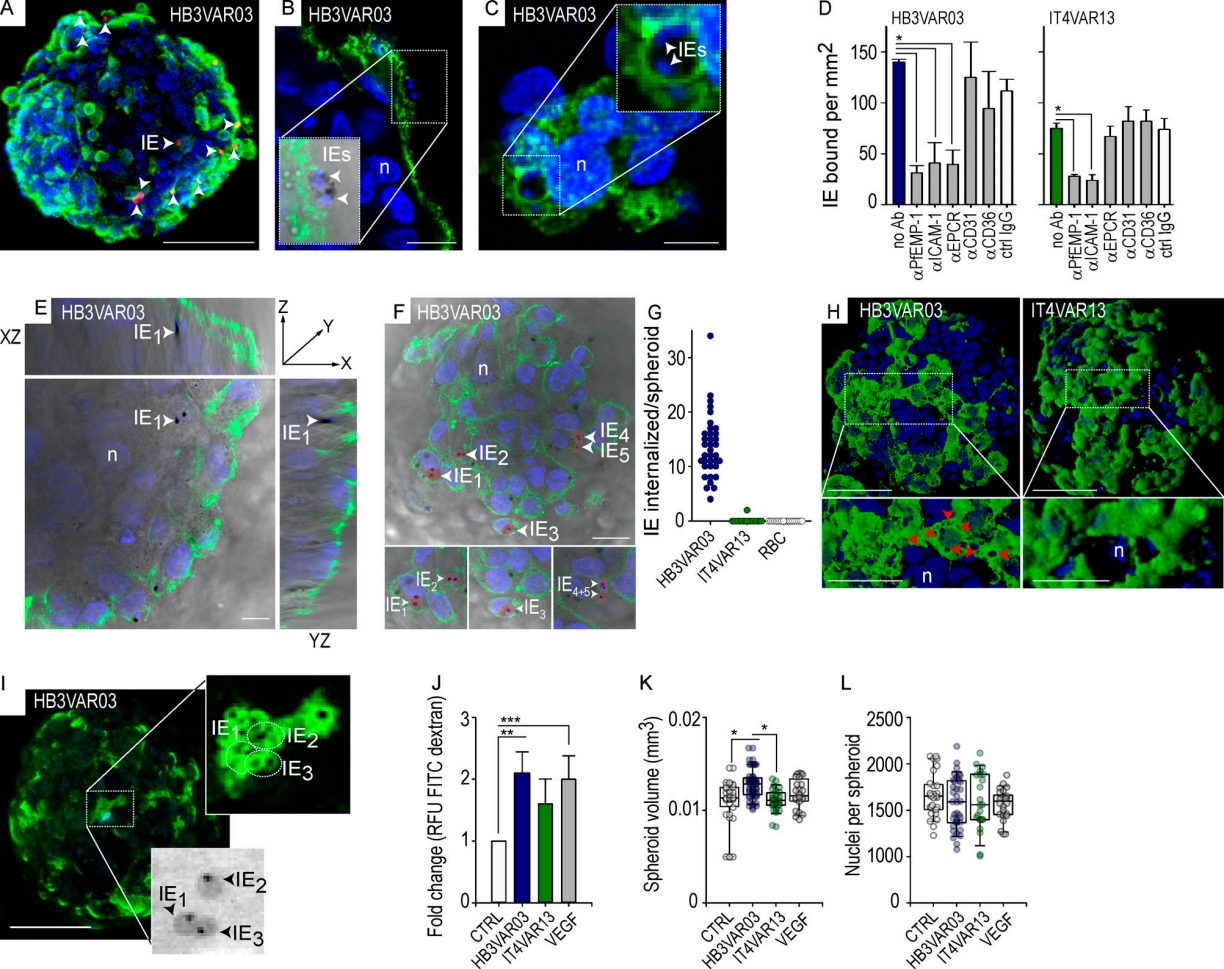
**Group A^+CM^ IE impact on the BBB.** **(A–C)** Confocal images representing a minimum of three independent experiments of BBB spheroids with adherent group A^+CM^ IEs (total number of spheroids, *n* = 309). **(A)** 3D z-projection of a spheroid showing PKH-26–stained IE (HB3VAR03) in red and ICAM-1 in green (Alexa 488). Scale bar, 100 µm. **(B)** Group A^+CM^ IEs (HB3VAR03) binding ICAM-1 (green) and in the process of entering the endothelium. Scale bar, 20 µm. **(C)** Group A^+CM^ IEs (HB3VAR03) surrounded by ICAM-1 (green). White arrowheads point to IEs. Scale bar, 10 µm. **(D)** Adhesion of group A^+CM^ (HB3VAR03) and B^+UM^ (IT4VAR13) IEs to BBB spheroids in the presence of antibodies against the homologous PfEMP1 (α-PfEMP1), ICAM-1 (α-ICAM-1), EPCR (α-EPCR), PECAM-1 (α-CD31), and CD36 (α-CD36). Shown are the mean number of IEs bound per square millimeter ± SD. All data represent a minimum of three independent experiments conducted in duplicate. Statistical analysis was done using one-way ANOVA with Tukey’s multiple comparison test (*, P < 0.001). Only significant findings are shown. **(E)** Representative orthogonal view of confocal image of HB3VAR03 group A^+CM^ IEs found below the endothelial cell surface. 3D projection from confocal z-stack; IE_1_ (white arrowhead) was found present in z-stack, shown is slice 23 of 57, which is 10.9 µm below the surface. White arrowhead points to the same IE (IE_1_) in all three panels. Scale bar, 20 µm. **(F)** Group A^+CM^ IEs (IE_1_–IE_5_) found below the endothelial cell surface. 3D projection from confocal z-stack. The white arrowheads point toward internalized group A^+CM^ IE (HB3VAR03) at a depth of 19.8 µm (slice 44 of 60; total number of spheroids, *n* = 79). Scale bar, 20 µm. **(G)** Group A^+CM^ IEs (HB3VAR03) but not group B^UM^ IE (IT4VAR13) are internalized by BBB spheroids. Spheroids with internalized group A^+CM^ IEs (HB3VAR03) and group B^UM^ IEs (IT4VAR13). Each data point represents a single spheroid and the number of IEs or noninfected erythrocytes (RBC, red blood cell controls) visualized by confocal microscopy beyond the ICAM-1 boundary after 8 h incubation (total number of spheroids, *n* = 76). **(H)** ICAM-1–enriched ring/docking structures induced by co-culturing of endothelial cells with group A^+CM^ HB3VAR03-IE (left). Co-culture with IT4VAR13 group B^UM^-IE does not induce such structures (right). Red arrowheads in the confocal image point to ring/docking structures; IEs are not visible in the two images (total number of spheroids, *n* = 309). ICAM-1 is green; nuclei stained with DAPI are blue. Scale bars, 100 µm. **(I)** Representative confocal image (z-projection) of spheroid with ring/docking structures induced by group A^+CM^ IEs (HB3VAR03). Ring/docking structures are indicated by dotted circles in top inset, and the IEs (IE_1_–IE_3_) found below the structures are shown in the brightfield image in the bottom inset. Black arrowheads points to IEs. Scale bar, 100 µm. **(J)** IE-induced alterations in barrier permeability measured as fold change ± SD in RFU-FITC dextran (70 kD) at 90 µm depth. VEGF (100 ng/ml) was used as a positive control. The data represent a minimum of three independent experiments (total number of spheroids, *n* = 140; 5–11 spheroids per group). Statistical analysis was done using one-way ANOVA with Tukey’s multiple comparison test (**, P = 0.001; ***, P = 0.0024). Only significant findings are shown. **(K)** Analysis of cell swelling. The spheroid volume (mm^3^) measured following coincubation with group A^+CM^ IE (HB3VAR03), group B^UM^ IE (IT4VAR13), and VEGF (100 ng/ml). The spheroids are the same as those in J. Statistical analysis was done using one-way ANOVA with Tukey’s multiple comparison test (*, P < 0.001). Only significant findings are shown. **(L)** Nuclei per spheroid following co-culture with group A^+CM^ IE (HB3VAR03), group B^UM^ IE (IT4VAR13), and VEGF (100 ng/ml; total number of spheroids, *n* = 140; 5–11 spheroids per group). Statistical analysis was done using one-way ANOVA with Tukey’s multiple comparison test; no statistical significant difference were found. Each data point in G, K, and L represents an individual spheroid. Ab, antibody; CTRL, control; n, nuclei.

**Figure S5.**
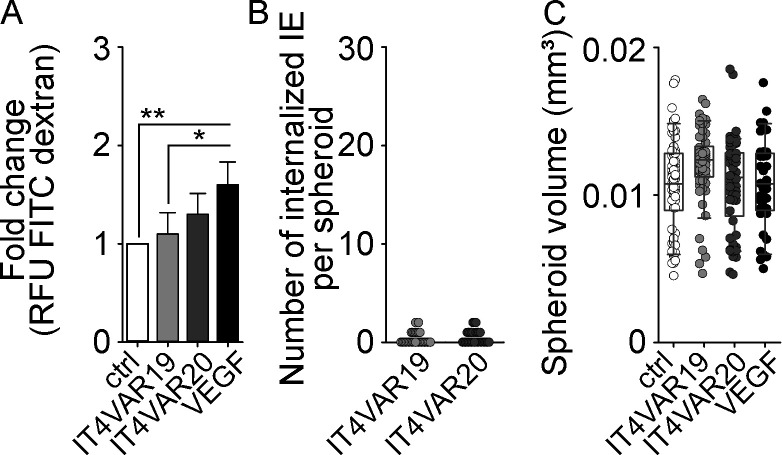
**Group B/A^SM^-IE binding EPCRs do not impact on the BBB. (A)** Bar chart showing the fold change in RFU of dextran-FITC uptake after overnight incubation with group B/A^SM^ IT4VAR19-IEs (gray) and group B/A^SM^ IT4VAR20-IEs (dark gray) compared with untreated control (white). VEGF (50 ng/ml) was added as a positive control for barrier disruption (black). **(B)** Dot plot showing number of internalized IEs per spheroid after coincubation with IT4VAR19-IEs (gray) and IT4VAR20-IEs (dark gray). Each circle represents an individual spheroid. **(C)** Measurements of volume (mm^3^) for each individual spheroid after exposure to IT4VAR19-IEs (gray), IT4VAR20-IEs (dark gray), VEGF (50 ng/ml; black), and compared with untreated controls (white). The data in all graphs are derived from four independent experiments (total number of spheroids, *n* = 302), and statistical significance was determined by one-way ANOVA (*, P = 0.0338; **, P = 0.0047). Only significant findings are indicated.

To investigate the effect of group A^+CM^ IEs on the BBB, a 3D spheroid model was employed (Cho et al., 2017). The spheroids, comprising astrocytes, pericytes, and endothelial cells, self-assemble within 48 h with a distinct cellular orientation, comparable to that of the BBB in vivo (Fig. S4, and as validated by Urich et al., 2013; Cho et al., 2017). Both group A^+CM^ HB3VAR03-IEs (Fig. 6, A–D) and group B^UM^ IT4VAR13-IEs (Fig. 6 D) bound spheroids after 1 h of coincubation. The adhesion of both parasite lines was abrogated by the addition of anti–ICAM-1 (clone 15.2) and antibodies targeting the DBLβ domain of PfEMP1 (Fig. 6 D). Addition of anti-EPCR–specific antibodies only inhibited the binding of dual ICAM-1/ECPR binding HB3VAR03-IEs. Extended co-culture (8 h) demonstrated spheroids internalizing group A^+CM^ HB3VAR03-IEs (Fig. 6, E–G), but not group B^UM^-IEs (IT4VAR13) or noninfected erythrocytes (Fig. 6 G). While the number of IEs internalized varied (4–34 IEs per spheroid), all spheroids exposed to group A^+CM^-IEs (HB3VAR03) internalized IEs (Fig. 6 G), with some observed at a depth of 10.9 and 19.8 µm (Fig. 6, E and F), indicating that the IEs had transmigrated the endothelial cells. Parasite-induced ICAM-1–rich ring/docking structures were also observed (Fig. 6 H, left), mimicking the observations from the monolayers (Fig. 1 A2 and Fig. 2 A). In some instances, group A^+CM^ (HB3VAR03) IEs colocalized (Fig. 6 I, bottom inset) with the ring/docking structures (Fig. 6 I, top inset). FITC-conjugated dextran sulfate was used to assess the permeability of spheroids, i.e., changes in barrier permeability. As shown by the significant increase (P = 0.001) in uptake of 70-kD dextran-FITC, exposure to group A^+CM^ HB3VAR03-IEs selectively altered the barrier integrity (Fig. 6 J). The dextran uptake was similar to that of the positive control, vascular endothelial growth factor (VEGF), known to disrupt endothelial barrier function (Wang et al., 2001). The barrier remained intact in the presence of group B^UM^ IT4VAR13-IEs and untreated controls (Fig. 6 J) as well as in the presence of group B/A^SM^ IT4VAR19- and IT4VAR20-IEs (Fig. S5 A). Another unexpected observation was the significant increase in the volume (Fig. 6 K) of spheroids co-cultured with group A^+CM^ IEs compared with the control and the group B^UM^ IT4VAR13 IEs (P < 0.001). Although it showed an impact on the barrier (Fig. 6 J), the VEGF control did not induce increased volume of spheroids (Fig. 6 K), indicating that the increased volume induced by group A^+CM^ IEs was due to cellular swelling and not influx of media. We observed no significant change in the number of nuclei (Fig. 6 L), indicating that increased spheroid volume was caused by cytosolic swelling as opposed to cell proliferation or influx of culture medium. Similar to IT4VAR13 group B^UM^ IEs (Fig. 6 G), the IT4VAR19 and IT4VAR20 group B/A^SM^ IEs did not become internalized (Fig. S5 B), and neither of the two different parasite-binding phenotypes altered the volume of spheroids significantly, or the number of observed nuclei (Fig. 6, K and L; and Fig. S5 C).

### Internalized IEs are observed in postmortem samples from fatal CM cases

**Figure 7.**
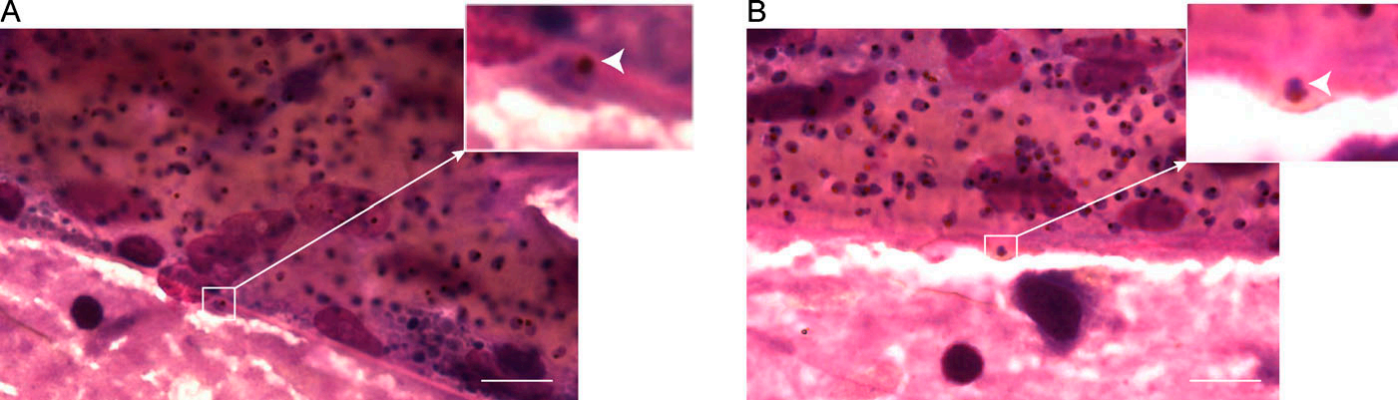
**Brightfield image of tissue from CM patients. (A and B)** May-Grünwald-Giemsa–stained postmortem brain samples from two different CM patients. Scale bars, 25 µm. Insets: Enlarged areas of interest showing IE internalized by endothelial cells. White arrows point to IEs. The assay was done blinded by two different highly experienced pathologists.

Biological relevance was confirmed in vivo using brightfield microscopy identifying the presence of internalized IEs in vivo from postmortem samples from two Indian CM patients (Fig. 7). The brain vasculature of such fatal CM cases showed vessels occluded by late-stage trophozoites. Crucially, and consistent with our other results, some endothelial cells in the patient tissue contained hemozoin pigment, and their plasma membranes appeared deformed and swollen compared with surrounding ones. Their membranes followed the outline of the mature IE, confirming internalization (Fig. 7). Thus, our findings identify entry and possible transmigration of endothelial cells as a property of *P. falciparum*–IEs expressing dual ICAM-1– and EPCR-binding group A PfEMP1s linked to CM (Lennartz et al., 2017).

## Discussion

Here we report for the first time the presence of intact, mature *P. falciparum* IEs within brain microvascular endothelial cells both in vitro and in vivo. A comparison of the endothelial responses to adhesion by well-defined parasite lines illustrated the differential effects elicited by group A^+CM^ IEs (ICAM-1– and EPCR-binding) expressing PfEMP1s with a highly conserved ICAM-1–binding sequence motif (Lennartz et al., 2017), as compared with group B^UM^ (ICAM-1– and CD36-binding or ICAM-1 only) and C^UM^ IEs (ICAM-1–binding), and group B/A^SM^ IEs (EPCR-binding). Interestingly, only group A^+CM^ IEs were observed to induce the clustering of surface-expressed ICAM-1, while the group B/A^SM^, group B^UM^, and group C^UM^ IEs expressing PfEMP1s without the motif did not. This finding leads us to propose that the endothelial cells act as nonprofessional phagocytic cells and respond to the adhesion of IEs expressing CM-associated PfEMP1s (group A^+CM^) by internalizing them. Once engulfed, the group A^+CM^ IEs slowly degrade, exposing the host cytoplasm to known toxins of endothelial cells, including hemozoin and cellular contents (Eugenin et al., 2019; Gallego-Delgado and Rodriguez, 2017; Griffith et al., 2009). Dual-receptor binding group A^+CM^ are capable of inducing ICAM-1 clustering via their motif-carrying DBLβ domain; this triggers internalization, likely by CAM-mediated endocytosis, a pathway involved in ICAM-1 recycling (Muro et al., 2005). By contrast, groups B^UM^, C^UM^, and B/A^SM^ IEs are apically retained. Combined with recent publications from nonmalaria groups (Gasimova, 2013; Fens et al., 2012), these observations of selective uptake of IEs by cerebral endothelial cells, BOEC, and primary HBMEC, and confirmed by human postmortem samples, show that the endothelium not only functions as a transport system for erythrocytes but also actively participates in erythrophagocytosis, removing from the bloodstream not only aged and damaged erythrocytes (Fens et al., 2012; Grutzendler, 2013) but also IEs expressing PfEMP1s linked to CM from the bloodstream. IE-induced receptor clustering has been reported previously, when Davis et al. (2013) demonstrated the recruitment of CD36 upon attachment of IEs expressing nongroup A PfEMP1s or protein/antibody-coated beads to HDMEC monolayers. However, while they clearly showed induction of CD36 clustering and rearrangement of the actin cytoskeleton, they reported no internalization of beads or IEs (Davis et al., 2013). In contrast, clustering of CD36 was associated with internalization by macrophages (McGilvray et al., 2000). The ICAM-1–rich, circular ring/docking structures observed on the endothelial cell surface after coincubation with group A^+CM^ IEs show a striking similarity with those induced by leukocytes. Leukocytes induce ICAM-1 clustering, resulting in the formation of transmigratory pore- or ring-like structures, which facilitate entry into the endothelium (Carman et al., 2003), and these docking structures have been shown to drive leukocyte internalization and transmigration through endothelial cells (Carman et al., 2003; Rahman and Fazal, 2009; Carman, 2009; Carman and Springer, 2004; Martinelli et al., 2013). The same receptor usage suggests that the mechanism is similar; however, as IEs lack the machinery necessary for motility, the transit of IEs is slower than that of leukocytes (Carman et al., 2003). ICAM-1 clustering has also been shown to drive the internalization of other pathogens such as rhinoviruses (Xing et al., 2003), *Listeria* spp. (Rengarajan et al., 2016), and *Bartonella* spp. (Dehio et al., 1997).

The selective removal and internalization of erythrocytes infected by *P. falciparum* expressing a particular subset of dual receptor binding group A^+CM^ PfEMP1s may confound existing inflammatory responses, resulting in disruption of the BBB and significant brain swelling as indicated by our data in Fig. 6, J and K. In addition to the detrimental exposure of parasite contents, the dual-adhesive IEs can interact with EPCR, which is expressed at low levels by the brain endothelium and can block the binding of activated protein C, leading to a loss of cytoprotection (Laszik et al., 1997; Moxon et al., 2013). Pre-treatment of endothelial cells with antibodies targeting ICAM-1 or EPCR showed that blockade of ICAM-1 inhibited this response, while an antibody targeting EPCR did not (Fig. 5 B). Thus, EPCR-binding IEs expressing group A^+CM^ PfEMP1s turn off cyto-protective responses via their CIDRα1 domain, while their ICAM-1–binding DBLβ domain induces clustering and a phagocytic response by the endothelium, contributing to CM pathology via the disruption of the BBB and cellular swelling. In contrast, control experiments with group B/A^SM^ EPCR-binding isolates (IT4VAR19 and 20) did not show significant disruption of the BBB or any significant internalization of IEs (Fig. S5, A and B). Previous literature has reported the ability of EPCR-binding isolates to disrupt the BBB; however, these studies were conducted using monolayers and with shorter 2 h assay times (Avril et al., 2019). The time chosen to measure disruption is crucial as thrombin-induced disruption of the BBB begins to repair itself after 30 min (Chen and Yeh, 2017). It is currently unknown how our findings translate into a physiological setting, and more studies on swelling and barrier integrity are needed to investigate this.

In conclusion, this is the first study showing human malaria asexual-stage parasites entering endothelial cells in vitro*.* Our ex vivo results (Fig. 7) are supported by one previous study reporting IE presence via postmortem tissue samples (Pongponratn et al., 2003). The results presented here underscore the need for a greater understanding of host–parasite interactions associated with specific binding phenotypes, but also a need to reassess our basic understanding of how IEs interact with the endothelium. Furthermore, our findings might have consequences for both the treatment of malaria and vaccine development. The role of the endothelium in this “accidental” clearance due to the utilization of ICAM-1 and the triggering of internalization pathways by group A^+CM^ IEs within 4 h of binding suggest that timely intervention is needed. Not all IEs are observed within endothelial cells, and given how slowly internalization occurs compared with leukocytes, this could explain why many parasites are still found in circulation in vivo*.* If a small proportion of internalized IEs has the potential to cause so much damage, it should be a priority to limit this CM pathology. Administration of thrombolytic drugs to treat stroke must be delivered swiftly after onset of symptoms to achieve the best outcome (National Institute of Neurological Disorders and Stroke rt-PA Stroke Study Group, 1995), and our data on the enhanced binding and internalization of IEs suggest that the same approach will be necessary to counteract not only the effects of cytoadhesion but also the subsequent contribution to potentially lethal brain swelling in CM.

## Materials and methods

### Malaria parasites

The *P. falciparum* lines 3D7 (PFD1235w), HB3 (VAR03, VAR21, and VAR34), BM057, and IT4 (VAR13) were maintained in in vitro blood culture and were selected using antibodies against DBLβ_D4 domains of specific PfEMP1s (Lennartz et al., 2017; Olsen et al., 2019; Bengtsson et al., 2013). IT4VAR19- and IT4VAR20-IEs were maintained in vitro blood cultures and selected for adhesion to EPCR by flowing parasite cultures (5–10% parasitemia) over EPCR (10 µg/ml)–coated ibidi slides (µ-Slide I 0.8 mm or µ-Slide VI 0.1 mm) for 20 min at a shear stress of 0.5 dyn/cm^2^. Non-bound IEs were removed by washing (RPMI-1640 plus 2% normal human serum, pH 7.2–7.4). Fresh parasite media (120 µl) were added to the channels and the slides placed into humidified Petri dishes at 37°C overnight. Following incubation, IEs were removed and transferred into fresh blood cultures; parasite growth was monitored by thin smear. Phenotypes were verified by flow cytometry and quantitative PCR as previously described (Lennartz et al., 2017; Olsen et al., 2019; Bengtsson et al., 2013; Lennartz et al., 2015; Joergensen et al., 2010).

### Human cell cultures

The human brain microvascular endothelial cell line hCMEC/D3 (CLU512; Cedarlane Labs) was maintained in collagen-coated (50 µg/ml; rat tail type I; BD Biosciences) flasks. The VascuLife VEGF-Mv media (LL0005; CellSystems) were supplemented with rhVEGF (recombinant human VEGF; 5 ng/ml), rhEGF (recombinant human epidermal growth factor; 5 ng/ml), rhFGF (recombinant human fibroblast growth factor; 5 ng/ml), rhIGF-1 (recombinant human insulin growth factor; 15 ng/ml), L-glutamine (10 mM), hydrocortisone hemisuccinate (1 µg/ml), heparin sulfate (0.75 U/ml), ascorbic acid (50 µg/ml), FBS (5%), 10,000 U/ml penicillin, 10,000 µg/ml streptomycin, and 25 µg/ml amphotericin B. Primary human brain microvascular pericytes and astrocytes (Neuromics) were maintained on poly-L-lysine (10 µg/ml)–coated flasks with pericyte (HMP104) and astrocyte (PGB003) growth media (Neuromics). Astrocytes and pericytes were used at passages 2–5 and hCMEC/D3 at passages 27–34. Primary HBMECs (Sciencell) were maintained on fibronectin (50 µg/ml; Millipore)–coated flasks and grown in endothelial cell media, supplemented with endothelial cell growth supplement (Sciencell). HBMECs were used at passages 2 and 3. BOECs were isolated from one European donor (Ecklu-Mensah et al., 2018) and maintained on collagen-coated (50 µg/ml; rat tail type I) flasks, and grown in endothelial growth medium-2 plus bullet kit (Lonza) supplemented with 10% FBS. BOECs were used at passages 2–4.

### BBB 3D spheroids

Human pericytes, astrocytes, and hCMEC/D3 released by 0.025% trypsin/EDTA (Sigma-Aldrich) were resuspended in BBB working medium (ScienCell 1001 supplemented with 2% normal human serum), and 1.5 × 10^3^ of each cell type (final volume of 100 µl/well) were seeded onto sterile 1% wt/vol solid agarose (Sigma-Aldrich) predispensed into low-binding 96-well plates (Thermo Fisher Scientific). A further 100 µl of BBB working media was added to bring the volume in each well to a total of 200 µl. Multicellular BBB spheroids were allowed to self-assemble (48–72 h) in a humidified 5% CO_2_ incubator at 37°C (Cho et al., 2017).

### Flow cytometry

*P. falciparum* IEs were DNA-labeled with ethidium bromide and surface-labeled with rat anti-PfEMP1 antibody (anti-HB3VAR21-DBLβ_D4 or anti-HB3VAR34-DBLβ_D4 antibodies; 1:20) and FITC-conjugated secondary rabbit anti-rat IgG (1:150; Vector Labs) as described previously (Joergensen et al., 2010). Fluorescence data from ethidium bromide–positive cells were collected on an FC500 MPL flow cytometer (Beckman Coulter) and analyzed using WinList, version 9.0 (Verity Software House). hCMEC/D3 and human brain microvascular pericytes were surface-labeled for CD31 and NG2, respectively. Human astrocytes were permeabilized with 0.1% Triton X-100 before staining with glial fibrillary acidic protein. Fluorescence data were collected on a CytoFlex S flow cytometer (Beckman Coulter) and analyzed using FlowJo (Becton Dickinson).

### Malaria parasite binding

For monolayer binding assays, hCMEC/D3 cells cultured in VascuLife VEGF-Mv supplemented as above were grown to confluence (3–4 d) on collagen-coated coverslips. Upon reaching confluence, cells were washed once with fresh, prewarmed adhesion media (1:1 mix of VascuLife VEGF-Mv and RPMI-1640, pH 7.4, 2% normal human serum). Trophozoite-stage parasites were adjusted to 3–5% parasitemia, and 300 µl of parasite suspension or noninfected O^+^ erythrocyte controls was added to each well and incubated (1–12 h) at 37°C. To remove nonbound cells, a minimum of four washes (500 µl/well) were done using prewarmed RPMI-1640. Coverslips were fixed with 2% vol/vol paraformaldehyde for 10 min at room temperature; blocking buffer (5% FBS/PBS plus 0.0025% Tween 20; 500 µl/well) was added before staining with anti–ICAM-1 (10 µg/ml; rabbit-polyclonal; SinoBiological).

Adhesion assays using viable BBB spheroids were done 72–96 h after assembly by adding trophozoite-stage parasites (100 µl/well; 3–5% parasitemia and 1% hematocrit) in BBB working medium. Following incubation with IEs at 37°C spheroids were transferred, washed in prewarmed RPMI-1640 to remove nonbound IEs, and then allowed to settle in low-bind Eppendorf tubes. Antibodies used were anti-PfEMP1 (1:100) antibody against HB3VAR03_DBLβ_D4 or IT4VAR13_DBLβ_D4, anti–ICAM-1 IgG (10 µg/ml; mouse clone 15.2; AbD Serotec), anti-EPCR IgG (10 µg/ml; goat polyclonal; R&D Systems), anti-CD31 IgG (10 µg/ml; mouse clone 9G11; R&D Systems), or isotype control IgG (10 µg/ml; mouse and goat; DAKO; or rat; Sigma-Aldrich). Cells and spheroids were incubated at 37°C for 1 h and then washed to remove unbound IEs. The surface of the spheroids was visualized by staining for ICAM-1 (10 µg/ml; anti–ICAM-1; SinoBiological) for 45 min, before washing and staining with anti-rabbit Alexa 488 (Invitrogen) secondary antibody for 45 min in the dark. Nuclei were visualized by DAPI (300 nM) stain. Images were recorded with Zeiss LSM 710 or LSM 780 confocal microscopes.

### Quantification of ICAM-1 expression around bound IEs

Human cerebral microvascular endothelial cells (hCMEC/D3) were grown to confluence on collagen (50 µg/ml)–coated coverslips. Trophozoite-stage IEs were added (3–5% parasitemia, 1% hematocrit; 300 µl in 1:1 mix of hCMEC/D3 media) and incubated at 37°C for 1 h. Non-bound cells were removed by 4 or 5 washes, and coverslips were fixed (2% vol/vol paraformaldehyde; 10 min) and blocked (500 µl/well; 5% FBS/PBS plus 0.0025% Tween 20) for 1 h at room temperature. ICAM-1 was visualized with anti–ICAM-1 (10 µg/ml; rabbit polyclonal; SinoBiological) and nuclei with 300 nM DAPI. The coverslips were imaged using a Zeiss LSM 780 scanning confocal microscope. Green channel images (ICAM-1 signal) were analyzed with Fiji using the line profile tool to draw a 20-µm line through the bound IEs, and the minimum/maximum relative fluorescence units (RFU) fold change was calculated along the profile. Control profiles were plotted using the same 20-µm distance at random areas across untreated cells. The difference between minimum/maximum was then expressed as fold change RFU compared with untreated controls. Quantification was done blinded and for at least 50 bound IEs per experiment, for a minimum of three independent experiments.

### Quantification of microvilli formation

hCMEC/D3 were grown and trophozoite-stage IEs were added as described above. Cells and IEs were co-cultured for 4–12 h, or overnight for noninfected red blood cell controls. Non-bound cells were removed by four or five washes, and coverslips fixed (4% vol/vol paraformaldehyde) and blocked as described above. ICAM-1 was visualized (45 min incubation in the dark) by anti-mouse IgG Alexa 488 (1:500; 100 µl 5% FBS/PBS plus 0.0025% Tween 20). PBS-washed coverslips were incubated with DAPI (300 nM; 5 min in the dark) to stain nuclei. This was followed by a final wash in distilled H_2_O. Coverslips were mounted with Vectashield anti-fade mounting medium. For each experiment, the number of cells with and without microvilli (40× magnification) was counted for a total of 500 endothelial cells using a Leica DMLB upright microscope. The researcher was blinded to the IE -sample, and the results were reported as percentage hCMEC/D3 cells with microvilli ± SD for a minimum of three independent experiments.

### Internalization of IEs by monolayers of human cells

Primary HBMEC, BOEC, and hCMEC/D3 were allowed to grow to confluence (3–4 d) on fibronectin- (50 µg/ml, HBMEC) or collagen- (50 µg/ml, BOEC and hCMEC/D3) pretreated coverslips. Trophozoite-stage IEs were added (3–5% parasitemia, 1% hematocrit; 300 µl in 1:1 mix of hCMEC/D3 media supplemented with 2% normal human serum and incubated at 37°C for 4 h (HBMEC and BOEC) or 8 h (hCMEC/D3). To block internalization (only hCMEC/D3), anti–ICAM-1 antibody (10 µg/ml; mouse clone 15.2; AbD Serotec) was added and incubated (1 h) in a humidified box at room temperature. Non-bound cells were removed by four or five washes, and coverslips were fixed (4% vol/vol paraformaldehyde; 10 min). ICAM-1 was visualized by staining with anti–ICAM-1 (10 µg/ml; rabbit polyclonal; SinoBiological) for 1 h at room temperature. Coverslips were then washed and incubated for 45 min with anti-rabbit IgG Alexa 488 (1:500; 100 µl 5% FBS/PBS plus 0.0025% Tween 20) in the dark. Coverslips were washed in PBS, then incubated with 300 nM DAPI (5 min in the dark), followed by a final wash in distilled H_2_O, and mounted with Vectashield anti-fade mounting medium. The researcher was blinded to the IE-sample, and coverslips were imaged using a Nikon Eclipse T2000, Zeiss LSM 710, or LSM 780 confocal microscope.

### Internalization of IEs by BBB spheroids

IEs (100 µl; HB3VAR03, IT4VAR13, IT4VAR19, and IT4VAR20) and BBB spheroids treated as above were coincubated for 8 h at 37°C, washed, and fixed for 10 min in 3.7% vol/vol formaldehyde. The spheroids were transferred to eight-well chamber slides (ibidi) following incubation with anti–ICAM-1 antibody and DAPI, and imaged with a Zeiss LSM 780 confocal microscope. Z-stacks were captured to 100-µm depth at 0.32 µm intervals (63×). Spheroid volumes (mm^3^) were calculated from (20×) brightfield images at 100 µm depth from diameter measurements generated in Fiji (Schindelin et al., 2012). To calculate volume, the following equation was used: *V* = 4/3π*r*^3^. Fiji particle analysis was used to calculate the number of nuclei per spheroid; all data were analyzed by one-way ANOVA with Tukey’s multiple comparisons test.

### Permeability assays

FITC-conjugated dextran sulfate (70 kD, 10 µg/ml) was used to assess the permeability of spheroids coincubated with HB3VAR03-IEs, IT4VAR13-IEs, IT4VAR19-IEs, IT4VAR20-IEs, or noninfected erythrocyte controls. Following overnight incubation with 100 µl of IEs (3–5% parasitemia, 1% hematocrit) or erythrocyte controls (1% hematocrit), spheroids were washed, then fixed with 3.7% vol/vol formaldehyde, their nuclei were stained with 300 nM DAPI, and they were transferred to 8-well chamber ibidi slides and z-stacks captured as described above. Using the green channel, the slice corresponding to 90 µm depth was selected, and a circle was drawn 50 µm from the outer border of the spheroid as previously reported (Cho et al., 2017). Using the integrated density, the RFU was measured for each spheroid and converted to fold change compared with untreated controls.

### Transmission electron microscopy

All transmission electron microscopy was done at the Core Facility for Integrated Microscopy at the University of Copenhagen. hCMEC/D3 cells were grown to confluence on Thermanox coverslips and then co-cultured (4, 8, 12, or 24 h) with HB3VAR03-IEs, IT4VAR13-IEs, or noninfected erythrocytes at 37°C. Non-bound cells were washed off, and the remaining cells were fixed with 2% vol/vol glutaraldehyde in 0.05 M sodium phosphate buffer (pH 7.2). Following isolation of suitable specimen blocks, the samples were rinsed three times in 0.15 M sodium phosphate buffer (pH 7.2) and subsequently post-fixed in 1% wt/vol OsO_4_ with 0.05 M K_3_Fe(CN)_6_ in 0.12 M sodium phosphate buffer (pH 7.2) for 2 h. The specimens were dehydrated in graded series of ethanol, transferred to propylene oxide, and embedded in Epon according to standard procedures. Sections, ∼60 nm thick, were cut with an Ultracut 7 (Leica) and collected on copper grids with Formvar supporting membranes, stained with uranyl acetate and lead citrate, and subsequently examined with a Philips CM 100 Transmission EM (Philips), operated at an accelerating voltage of 80 kV. Digital images were recorded with an OSIS Veleta digital slow scan 2k × 2k CCD camera and the ITEM software package.

### Brain smears

Brain samples were collected from two fatal adult CM cases as part of an ongoing study at Ispat General Hospital, India, as approved by the institutional review boards of Ispat General Hospital, the Government of India, New York University School of Medicine, and the London School of Hygiene and Tropical Medicine. After consent was obtained from the families, supraorbital brain sampling was performed as described elsewhere (Milner et al., 2005). Standard brain smears were then prepared (Brownell, 1981), air-dried, stained with May-Grünwald-Giemsa, and mounted. Slides were examined using a brightfield Leica Leitz DMRB microscope (100× oil immersion) and digital images acquired with a Retiga 2000R camera operated by Volocity software suite v6.3. The two expert pathologists who assessed the slides were blinded to what they were analyzing and asked to make observations.

### Statistical analysis

IE binding levels reported in Fig. 1 and Fig. 6, IE internalization data in Fig. 5, Fig. 6, and Fig. S5, microvilli data in Fig. 2 and Fig. S3, and permeability experiments in Fig. 6 and Fig. S5 are derived from a minimum of three independent experiments, ±SD. Statistical analysis was determined by performing an unpaired *t* test or one-way ANOVA with Tukey’s multiple comparison test as indicated. All quantitative assays were done blinded to the researcher.

### Online supplemental material

Fig. S1 shows the domain structure of group A, B, and C PfEMP1 proteins, their receptor adhesive properties, and association with cerebral, severe, or mild malaria. Fig. S2 shows the *var* gene and PfEMP1 expression profile of HB3VAR21 and HB3VAR34, newly identified ICAM-1–binding *P. falciparum* parasite lines used in this study. Fig. S3 shows the levels of microvilli production by hCMEC/D3 endothelial cells induced by parasite exposure (i.e., group A^+CM^ [3D7 PFD1235w and BM057] dual ICAM-1/EPCR-binding IEs, and group B^UM^ [HB3VAR21] and group C^UM^ [HB3VAR34] IEs). Fig. S4 shows the composition of the 3D BBB spheroids and surface profile of cell-specific markers of component cells of the BBB. Fig. S5 shows the measurements of profile barrier integrity, volume, and IE internalization by group B/A^SM^ IT4VAR19 and IT4VAR20 EPCR binding IEs.
